# Impact of Condition Variations on Bioelectrochemical System Performance: An Experimental Investigation of Sulfamethoxazole Degradation

**DOI:** 10.3390/molecules29102276

**Published:** 2024-05-12

**Authors:** Qun Xue, Zhihui Chen, Wenjing Xie, Shuke Zhang, Jie Jiang, Guoxin Sun

**Affiliations:** 1 College of Environmental Science and Engineering, Beijing Forestry University, Beijing 100091, China; xq1013942152@163.com (Q.X.); ythy0002267@163.com (Z.C.); xwj5037@163.com (W.X.); 17865567961@163.com (S.Z.); 2 State Key Lab of Urban and Regional Ecology, Research Center for Eco-Environmental Sciences, Chinese Academy of Sciences, Beijing 100085, China

**Keywords:** bioelectrochemical system, electrolysis cell, H-type cell, sulfamethoxazole, *Shewanella oneidensis* MR-1

## Abstract

Bioelectrochemical systems (BESs) are an innovative technology for the efficient degradation of antibiotics. *Shewanella oneidensis* (*S. oneidensis*) MR-1 plays a pivotal role in degrading sulfamethoxazole (SMX) in BESs. Our study investigated the effect of BES conditions on SMX degradation, focusing on microbial activity. The results revealed that BESs operating with a 0.05 M electrolyte concentration and 2 mA/cm^2^ current density outperformed electrolysis cells (ECs). Additionally, higher electrolyte concentrations and elevated current density reduced SMX degradation efficiency. The presence of nutrients had minimal effect on the growth of *S. oneidensis* MR-1 in BESs; it indicates that *S. oneidensis* MR-1 can degrade SMX without nutrients in a short period of time. We also highlighted the significance of mass transfer between the cathode and anode. Limiting mass transfer at a 10 cm electrode distance enhanced *S. oneidensis* MR-1 activity and BES performance. In summary, this study reveals the complex interaction of factors affecting the efficiency of BES degradation of antibiotics and provides support for environmental pollution control.

## 1. Introduction

Sulfamethoxazole (SMX) was the earliest synthetic drug for the prevention and treatment of bacterial infections, and its low production cost and broad-spectrum activity [1,2] make it widely used in aquaculture [3]. However, because of its low water solubility and resistance to degradation, its effective removal poses a challenge, even in wastewater treatment plants [4,5]. Moreover, many breeding areas lack basic measures for removal, leading to significant direct discharge of SMX-contaminated wastewater into surface waters. The ongoing rise in SMX use in low- and middle-income countries and highly populated regions has led to global concern regarding the high concentration of SMX in wastewater [6].

The presence of residual antibiotics in the environment not only inhibits the growth and activities of microorganisms, but also poses a threat to human health while destroying ecological functions and threatening the stability of the ecosystem [7,8,9]. SMX, for instance, exhibits chronic toxic effects and can cause chronic poisoning symptoms. SMX can disturb the body’s beneficial bacteria and cause dysbiosis of the gut microbiome with long-term use, boosting the production of antibiotic-resistant genes among microorganisms [10,11,12]. Additionally, SMX can induce toxic effects in animals and plants while promoting microbial-resistance genes to spread rapidly across the environment, humans, and animals [10,13,14]. As such, efficient, economical, and simple treatment technologies are essential in addressing high-concentration fishery wastewater containing SMX.

The main dissipation pathway of SMX in engineering systems and in nature is biodegradation. Affected by various influencing factors, the time activated sludge takes to degrade SMX in full-scale wastewater treatment plants is not stable, ranging from a few hours to dozens of days, and the degradation effect varies greatly [15]. Using biodegradation techniques, SMX can be degraded initially by S–N bond cleavage [16]. *Shewanella oneidensis* (*S. oneidensis*) MR-1, which belongs to the *Shewanella* group, has the unique ability to utilize SMX as a carbon source and energy for growth; it is often used to study the biodegradation of SMX [17,18].

Electrochemical remediation is an advanced technology for in situ treatment of aquaculture wastewater [19]. It has been found that electrochemistry can remove SMX from wastewater through the exchange of electrons with the redox-active electrons produced by the cathode and anode, and the SMX removal is mainly dependent on the electrogenerated hydroxyl radicals during electrolysis [20,21,22,23]. Despite its high removal efficiency, electrochemical technology is limited by its oxidative efficiency in wastewater treatment [24,25]. Thus, researchers have focused on developing advanced electrodes, such as graphene or boron-doped diamond electrodes, that exhibit higher oxidation potentials [26,27]. However, the high cost of these electrodes and the complex manufacturing processes present significant obstacles to their use in practical applications [28].

The combination of electrochemistry and microbial technology has promoted the development of bioelectrochemical systems (BESs) [29,30,31]. BESs primarily consist of three different operation modes. The microbial fuel cell (MFC) features a biological anode and abiotic cathode, and microorganisms utilize antibiotics as electron donors and carbon sources in the anode for degradation. Another BES with a structure similar to MFCs utilizes modified materials to generate hydroxyl radicals in the abiotic cathode which then attack antibiotics for degradation. In microbial electrolysis cells (MECs), where microorganisms are present at both the cathode and anode and have an external power supply, the degradation of antibiotics is typically achieved through direct electrochemical reduction as well as biodegradation [32]. However, in BESs, microbial viability is crucial and typically requires the presence of a biological film as an attachment, which leads to a long process to effectively remove SMX [33,34].

*Shewanella* has become an important organism in the study of bioelectrochemical processes because of its special electroactivity ability. However, there is currently limited research on the effectiveness of *S. oneidensis* MR-1 for the electrochemical degradation of antibiotics, especially in high-concentration antibiotic wastewater. Parameters such as current, electrolyte concentration, and electrode distance in the electrochemical degradation scheme can affect the activity and function of microorganisms in the bioelectrochemical process [25,27].

In response to the widespread problem of antibiotic residues in the ecosystem, measures to separate urine at source are being used to reduce the flow of drugs into centralized wastewater treatment plants and their release into the environment. Concentrations of antibiotics in source-isolated urine and wastewater treatment plants are significantly higher than in the environment, and the importance of efficient treatment of high concentrations of antibiotics is becoming increasingly apparent. The purpose of this study is to investigate the effects of electrodynamic repair parameters such as electrolyte composition, current density, and electrode distance on microbial growth in BESs over a short period of time so as to explore the effects of reaction parameters on the efficiency of the degradation of SMX by biofilm-free BESs. By combining the advantages of electrochemical degradation and microbial degradation, a BES is proposed to degrade high-concentration SMX in a short time.

## 2. Results and Discussion

### 2.1. Reaction Process in Bioelectrochemical Systems

**Figure 1:**
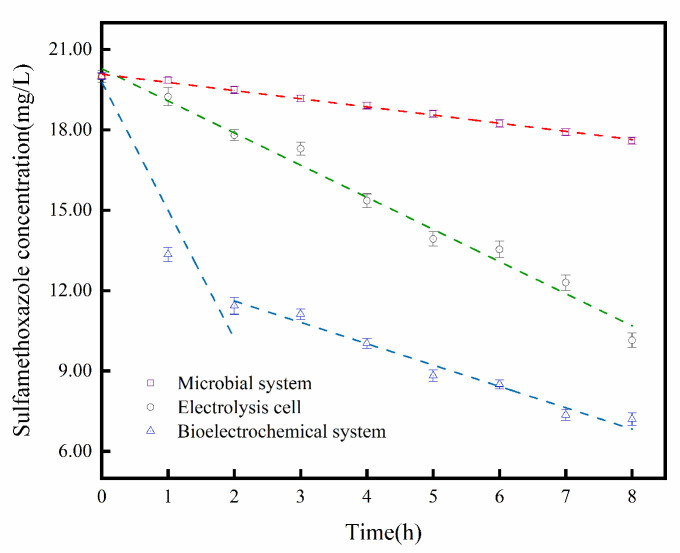
Concentration variation of SMX in three different systems.

In this study, we first compared the degradation of SMX by three systems within eight hours. The initial concentration of SMX was 20 mg/L. The microbial system contained only MR-1, and the electrochemical system was an electrolysis cell (EC). The BES was made by adding *S. oneidensis* MR-1 to an EC. The supplement solution of the microbial system and BES was LB (Luria–Bertani) medium. The electrochemical parameters of the BES were consistent with those of the electrochemical system. The SMX degradation efficiency in the microbial system was observed to be 12% over an 8 h period. The EC and BES displayed removal efficiencies of 49.25% and 64%, respectively (Figure 1).

The time curve of SMX degradation in the BES exhibits that, during the first two hours of the reaction, the concentration of SMX in the solution exhibited a sharp and rapid decline. This initial phase is characterized by the high activity of *S. oneidensis* MR-1, which was widely distributed in the BES. Because the voltage stimulated the activity of *S. oneidensis* MR-1 [35], the degradation efficiency of the BES was greater than the sum of biodegradation and electrochemical degradation.

However, from the third hour onwards, the degradation curve of SMX gradually flattened. At the same time, the activity of *S. oneidensis* MR-1 during the reaction gradually decreased; it reduced the promotion of SMX degradation. In the later stages of the reaction, inactive *S. oneidensis* MR-1 and the metabolites in the system hinder the mass transfer rate, thereby impeding SMX from accepting electrons and consequently leading to the ineffective degradation of SMX.

### 2.2. Impact of Solution Matrix

To maintain the activity and functionality of *S. oneidensis* MR-1, LB medium was added to the reactor as a nutrient source during the experiments. However, the medium contains various substances, contributing to the complexity of the ions around the electrodes [36]. Considering that both SMX and ECs can serve as energy sources for *S. oneidensis* MR-1 growth [25], ultrapure water and LB medium were employed to investigate the impact of the medium on the real-time activity of *S. oneidensis* MR-1 and SMX degradation.

**Figure 2:**
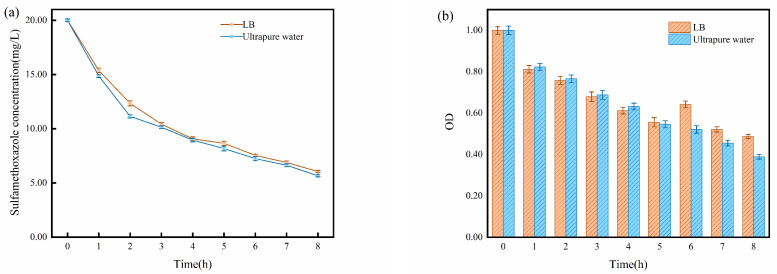
(**a**) Degradation of SMX under different supplementations (LB medium, ultrapure water); (**b**) OD variation of *S. oneidensis* MR-1 under different supplementations (LB medium, ultrapure water).

The trend and efficiency were similar in both reactors. However, the reactor supplemented with ultrapure water showed slightly better SMX degradation compared to the one with LB medium (Figure 2a). This phenomenon might be attributed to the interference caused by the complex substances in the LB medium near the electrodes. These substances could potentially compete with SMX for degradation, leading to a minor hindrance in the SMX degradation process.

In this study, we use optical density (OD) values to represent the activity of *S. oneidensis* MR-1. As for the OD variation, in the early stages of the reaction, *S. oneidensis* MR-1 exhibited higher OD in ultrapure water, whereas, in the later stages, its OD in LB medium surpassed that in ultrapure water (Figure 2b). The concentration of SMX decreased in the later stage of the reaction. In this case, compared to ultrapure water, LB medium provided more nutrients required for microbial proliferation and better supported the growth of *S. oneidensis* MR-1 at this stage.

Previous studies have demonstrated that the efficiency of the removal of pollutants in the EC is directly proportional to the concentration of electrolytes within a certain range [25]. This correlation can be attributed to the increased ion strength resulting from the addition of inorganic salts, which facilitates the transfer of organic pollutants to the gas–liquid interface and alters vapor pressure and surface tension, thereby promoting the formation of bubbles in the solution [37]. Consequently, these effects enhance the removal of pollutants from the system. In light of the correlation between electrolyte concentration and pollutant removal in the EC, further investigations have been conducted to explore the influence of varying electrolyte concentrations on the performance of BESs.

**Figure 3:**
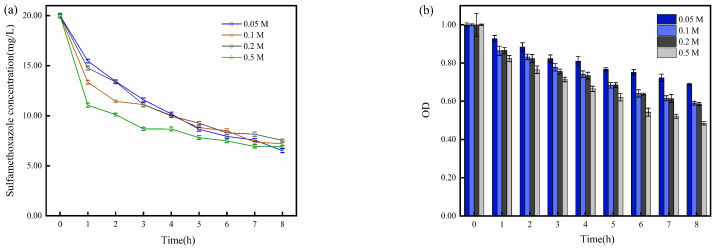
(**a**) Degradation of SMX at different electrolyte concentrations (0.05, 0.1, 0.2, 0.5 M); (**b**) OD variation of *S. oneidensis* MR-1 at different electrolyte concentrations (0.05, 0.1, 0.2, 0.5 M).

As shown in Figure 3a, during the first hour, the degradation rate of SMX in the 0.5 M electrolyte system was significantly higher than that in the 0.05 M electrolyte system. However, at the eighth hour, the SMX degradation rate in the 0.5 M electrolyte system was slightly lower than that in the 0.05 M electrolyte system. Concurrently, based on the OD changes presented in Figure 3b, it is evident that, at the eighth hour, the OD value of *S. oneidensis* MR-1 in the 0.5 M electrolyte system was substantially lower than that in the 0.05 M system. This observation suggests that the higher concentration of 0.5 M electrolyte exerts a strong inhibitory effect on *S. oneidensis* MR-1 activity, while the relatively lower concentration of 0.05 M electrolyte is more conducive to microbial growth. Although the 0.5 M electrolyte concentration diminishes microbial activity, it concurrently promotes the electrochemical degradation of SMX. Consequently, the final efficiency of the degradation of SMX showed a slight variation between these two systems.

In conclusion, LB medium does not significantly enhance *S. oneidensis* MR-1 activity in BESs. Moreover, the inclusion of LB medium introduces complex substances that may negatively impact the degradation of SMX. Additionally, high electrolyte concentrations can inhibit *S. oneidensis* MR-1 activity, leading to potential performance issues in BESs. In contrast, lower electrolyte concentrations are more conducive to *S. oneidensis* MR-1 growth, leading to better degradation performance.

### 2.3. Impact of Current Density

Previous studies have shown that currents ranging from 15 μA/cm^2^ to 20 mA/cm^2^ can stimulate microbial activity in the environment [38,39], thereby enhancing the microbial degradation of pollutants. However, the direct microbial removal of pollutants still requires several days to weeks. As demonstrated in Section 2.1, BESs can effectively remove SMX within 8 h. To investigate the impact of different current densities on SMX removal in BESs, four current densities of 2, 5, 10, and 20 mA/cm^2^ were set in the experiment.

**Figure 4:**
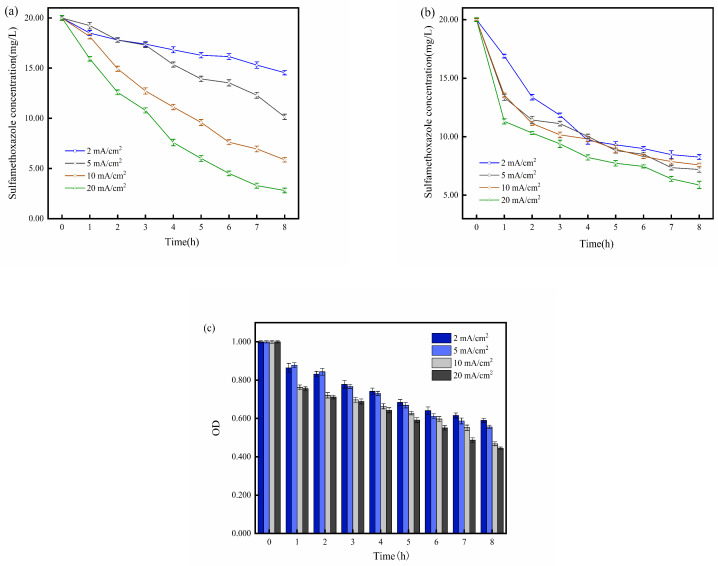
(**a**) Degradation of SMX at different current densities in electrolysis cell (2, 5, 10, 20 mA/cm^2^); (**b**) degradation of SMX at different current densities in bioelectrochemical system (2, 5, 10, 20 mA/cm^2^); (**c**) OD variation of *S. oneidensis* MR-1 at different current densities (2, 5, 10, 20 mA/cm^2^).

As the current density increased, the degradation of SMX in the EC improved (Figure 4a). This observation aligns with previous research [25]. The experimental results for the BES, as shown in Figure 4b, indicate that, within the first two hours of the reaction, microorganisms significantly promoted SMX degradation under different current density conditions. However, from the third hour onwards, the degradation curve of SMX gradually flattened, and the differences in degradation rates among the four current density conditions became less pronounced. After three hours, the BES demonstrated a reduced degradation rate of SMX under high current density relative to the EC. The differences in the final SMX degradation efficiency under different current density conditions in the BESs were significantly reduced compared to the ECs. When the current density in the system exceeds 10 mA/cm^2^, the SMX degradation efficiency of the BES becomes inferior to that of the EC. However, at current densities below 10 mA/cm^2^, adding microorganisms has a pronounced promoting effect on SMX degradation. Consistent with the above phenomenon, the OD values of *S. oneidensis* MR-1 at current densities of 10 and 20 mA/cm^2^ are significantly lower than those of 2 and 5 mA/cm^2^ (Figure 4c). The reason behind this phenomenon is that low current density can enhance microbial activity, diversity, and electrophoretic speed, thereby improving microbial degradation capabilities [40]. On the other hand, high current density not only increases microbial cell surface hydrophobicity and flattens cells but also increases extracellular substances and net surface negative charge on microbial cells, causing damage to microbial activity and functional diversity [41,42]. Therefore, in the BES, although high current density can improve electron transfer efficiency within the system, it can also result in significant damage to microorganisms, leading to many inactive microorganisms in the reactor during the later stages of the reaction, which may hinder pollutant degradation.

### 2.4. Impact of Electrode Distance

In the electrochemical degradation process, electrode distance is also a crucial factor influencing the degradation efficiency. In ECs, the degradation efficiency is inversely proportional to the electrode distance. As the distance between the electrodes increases, the degradation efficiency decreases accordingly [43,44]. This is primarily due to the increased circuit length of the electrode, liquid surface, and external power supply, leading to higher resistance and reduced current migration rate. Additionally, as the electrode distance increases, the distance for substances to migrate between the anode and cathode also increases, resulting in a decrease in mass transfer rate. The following experiments further confirmed this conclusion, where the electrode distance was set to 10 cm in an H-type EC. The H-type EC reduced the cross-sectional area between the anode and cathode, further limiting the substance transfer.

**Figure 5:**
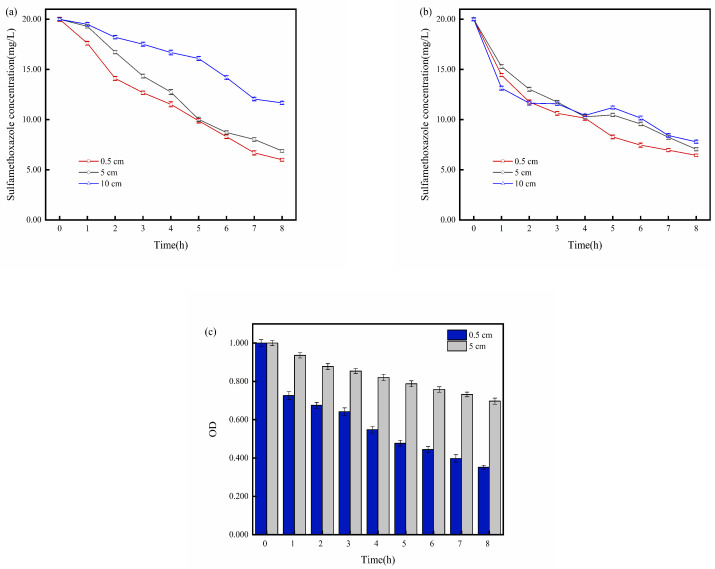
(**a**) Degradation of SMX at different electrode distances in electrolysis cell (0.5, 5, 10 cm); (**b**) degradation of SMX at different electrode distances in bioelectrochemical system (0.5, 5, 10 cm); (**c**) OD variation of *S. oneidensis* MR-1 at different electrode distances (0.5, 5, 10 cm).

As shown in Figure 5a, the efficiency of the electrochemical degradation of SMX in the EC with an electrode distance of 10 cm was significantly lower than in the cells with electrode distances of 0.5 cm and 5 cm. In the BESs with different electrode distances corresponding to ECs, SMX degradation was accelerated to varying degrees in the early stages of the reaction. As the reaction progressed, the concentration of SMX continued to decrease. After 8 h, the BES with an electrode distance of 10 cm exhibited significantly improved final SMX degradation compared to the EC, while residual concentrations of SMX after 8 h in 0.5 cm and 5 cm BESs were slightly higher than in ECs (Figure 5b). The deterioration of the degradation could be because, in the later stage of the reaction, the OD values of *S. oneidensis* MR-1 decreased (Figure 5c). The increased inactive *S. oneidensis* MR-1 in the cells hindered the electron transfer between the electrode and SMX.

**Figure 6:**
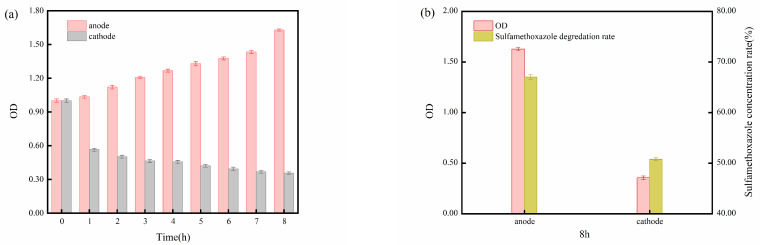
(**a**) OD variation of *S. oneidensis* MR-1 around two different electrodes in H-type electrolysis cell within 8 h; (**b**) *S. oneidensis* MR-1 OD and SMX degradation rates around two different electrodes at the 8th hour.

As shown in Figure 5c, active *S. oneidensis* MR-1 in the BES with 0.5 cm electrode distance decreased faster than in those with 5 cm electrode distance, which indicates that the longer electrode distance provides more space with suitable redox conditions for *S. oneidensis* MR-1 growth. Additionally, due to the limitations imposed by the H-type reactor on substance transfer, the environment around the two electrodes underwent different changes during the reaction process. The solution near the anode gradually became transparent, showing a pale pink color, while the solution near the cathode turned into an opaque milky white. Combining the OD data of the solutions near both electrodes in Figure 6a, it is evident that the microbial concentration near the anode gradually increased during the experimental process. In contrast, the concentration of active microorganisms near the cathode decreased progressively. The observed increase in the OD value near the anode during the later stages of the reaction, contrary to the previous experimental trend of declining OD values, can be attributed to the limitations on mass transfer imposed by the H-type reactor design, which resulted in a relatively stable environment for *S. oneidensis* MR-1 near the anode. Therefore, under the stimulation of a positive charge at the anode, the microbial concentration near the anode continuously grew and reproduced during the reaction. On the other hand, the negative charge of the cathode inhibited the activity of *S. oneidensis* MR-1, resulting in the gradual deactivation of *S. oneidensis* MR-1 near the cathode as the reaction proceeded. Consequently, a significant accumulation of deactivated *S. oneidensis* MR-1 and its metabolites near the cathode caused the solution to be turbid.

The final degradation efficiency of SMX near the two electrodes in the H-type reactor also exhibited differences, with the anode exhibiting an 8 h degradation rate of 67% and the cathode showing a rate of 51% (Figure 6b). The degradation of SMX near the anode is mainly dependent on *S. oneidensis* MR-1, which uses SMX as a carbon and nitrogen source [15], while SMX at the cathode obtains electrons from the cathode and is directly reduced by electrochemical reduction [31]. These results re-emphasized the significance of microorganisms in BESs, particularly when the electrode distance is relatively large. When the distance between electrodes is substantial and the mass transfer rate is slow, the SMX located at a greater distance from the electrodes cannot be effectively degraded. In the same way, when the electrode distance is short, the range of pollution space that ECs can treat is also limited, highlighting the limitations of ECs in engineering applications. The addition of *S. oneidensis* MR-1 and its aggregation near the anode allow for SMX degradation at the previously non-reactive anode, thereby compensating for the limitation of ECs in engineering applications.

## 3. Materials and Methods

### 3.1. Materials

The pollutant used for the experiment was 99.9% SMX, sourced from Shanghai Aladdin Biochemical Technology Co., Ltd. (Shanghai, China). The electrolyte employed was analytical-grade sodium sulfate, which was also obtained from the same supplier. Sodium chloride was procured from Shanghai Merck Chemical Technology Co., Ltd. (Shanghai, China), and peptone and yeast extract were obtained from Oxoid, based in the Altrincham, UK. Agar was purchased from Shanghai Aladdin Biochemical Technology Co., Ltd. (Shanghai, China). Chromatography-grade methanol, acetonitrile, and acetic acid were obtained from Shanghai Merck Chemical Technology Co., Ltd. (Shanghai, China). All other reagents except culture media were prepared using ultrapure water. *S. oneidensis* MR-1 was initially isolated from the Chinese Academy of Sciences Research Center for Eco-Environmental Sciences and has since been maintained in culture by ourselves.

### 3.2. Microbiological Culture

Preparation of LB medium: NaCl 5 g, yeast extract 2.5 g, and tryptone 5 g. Combine these ingredients in a 1 L beaker with 500 mL of deionized water and stir with a glass rod until fully dissolved. Distribute the solution evenly into five conical flasks, seal them with aluminum foil, and autoclave at 121 °C for 30 min for sterilization.

Preparation of *S. oneidensis* MR-1 solution: One bacterial colony selected from a solid culture using a sterile inoculation loop was inoculated into a sterile LB medium. The mixture was covered with aluminum foil to prevent contamination and incubated at a shaking speed of 220 rpm for 16–19 h in darkness at 30 °C.

### 3.3. Reactor Setup and Experimental Procedure

**Figure 7:**
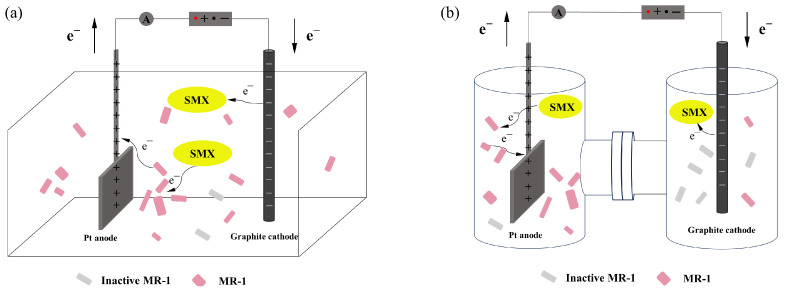
Schematic diagram of bioelectrochemical systems. (**a**) Standard cell; (**b**) H-type cell.

The experimental setup is shown in Figure 7. The internal dimensions of the standard cell were 10 cm length, 5 cm width, and 7 cm height (Figure 7a). The single cell of the H-type EC has a diameter of 5 cm and a height of 7 cm. The length of the connecting channel is 4 cm (Figure 7b). The anode was made of a platinum-plated titanium electrode, sized 2 × 2 × 0.2 cm^3^. The cathode was a high-purity graphite electrode, 15 cm high and with a diameter of 0.8 cm. Other supporting devices included an adjustable direct current power supply, a milliampere meter, and connecting wires.

The experimental design included variations in solute, current density, and electrode distance. The solute variations of the reaction solution included supplementation types and electrolyte concentrations. The experiments were conducted using the single-factor method. The details of variables investigated were supplementations (LB medium, ultrapure water), electrolyte concentration (0.05, 0.1, 0.2, 0.5 M), current density (2, 5, 10, 20 mA/cm^2^), electrode distance (0.5, 5, 10 cm).

Experiments were conducted within an anaerobic chamber to maintain controlled environmental conditions. The anaerobic chamber underwent three cycles of vacuuming and nitrogen gas filling to ensure an oxygen-free environment. The EC was prepared by introducing SMX solution, sodium sulfate electrolyte, and supplementation, ensuring an initial SMX concentration of 20 mg/L. Electrodes were accurately installed according to set distances and connected to the power source. The experiments with electrode distances of 0.5 cm and 5 cm were conducted in the EC depicted in Figure 7a, while the experiment with an electrode distance of 10 cm was conducted in the EC depicted in Figure 7b. The experimental procedure commenced when the system was energized and *S. oneidensis* MR-1 was added to the EC, reaching an OD of 1 at the same time. Subsequently, the degradation effect of BESs without biofilm on high concentrations of SMX was explored over an 8 h period.

### 3.4. Sampling and Analyses

The reaction time was set for eight hours, and samples were collected every hour from the anode, cathode, and mixed electrode solution. OD value and SMX concentration were measured respectively. The OD measurement does not require pre-processing, and the SMX measurement requires centrifuging the sample at 14,800 rpm for 5 minutes followed by filtration through a 13 mm, 0.22 μm aqueous filter membrane and storage at −20 °C. 

The OD value of *S. oneidensis* MR-1was measured at a wavelength of 600 nm. Three 200 μL samples were added to the wells of the 96-well microplate for determination. The average OD values of 3 samples were taken as the actual OD values of *S. oneidensis* MR-1 in the sample.

SMX was detected with the Agilent 1260 liquid chromatography system. The chromatography was performed on Agilent Pinnacle II C18 (5 μm, 4.6 × 150 mm) column at 30 °C. The sample size was 10 μL, and the detection time was 7 min. The eluent was composed of acetonitrile and 0.4% acetic acid at a ratio of 7:3 with a flow rate of 1 mL/min.

## 4. Conclusions

In BESs that do not contain biofilms, the presence of microorganisms rapidly improves the ability of ECs to degrade SMX, effectively degrading high concentrations of SMX within 8 h. The results of the study on the influence parameters of BESs showed that LB medium had little effect on the growth of *S. oneidensis* MR-1, and the nutrient influence was limited. High electrolyte concentration will inhibit MR-1 activity and reduce degradation efficiency. Increasing the current density also reduces the promoting effect of MR-1 and slows the degradation of SMX. In practical application, parameters such as electrolyte concentration and current density should be adjusted to ensure early microbial activity. The high activity of *S. oneidensis* MR-1 on distant electrodes with slow mass transfer rates and the excellent degradation of SMX at high concentrations underscore the engineering potential of BESs. BESs without biofilm can rapidly degrade source-isolated wastewater containing a high concentration of antibiotics without acclimation.

## Data Availability

All data are included in the manuscript, and all results of this study are available from the corresponding author.
